# Success rates of simulated multi-pulse defibrillation protocols are sensitive to application timing with individual, protocol-specific optimal timings

**DOI:** 10.3389/fnetp.2025.1572834

**Published:** 2025-05-27

**Authors:** Marcel Aron, Stefan Luther, Ulrich Parlitz

**Affiliations:** ^1^ Biomedical Physics Group, Max Planck Institute for Dynamics and Self-Organization, Göttingen, Germany; ^2^ Institute for the Dynamics of Complex Systems, Georg-August-Universität Göttingen, Göttingen, Germany; ^3^ German Center for Cardiovascular Research (DZHK), Partner Site Lower Saxony, Göttingen, Germany; ^4^ Cluster of Excellence “Multiscale Bioimaging: From Molecular Machines to Networks of Excitable Cells” (MBExC), University of Göttingen, Göttingen, Germany; ^5^ Institute of Pharmacology and Toxicology, University Medical Center Göttingen, Göttingen, Germany

**Keywords:** cardiac arrhythmia, ventricular fibrillation, low-energy defibrillation, excitable media, chaos control, network physiology

## Abstract

Ventricular fibrillation is a lethal condition where the heartbeat becomes too disorganised to maintain proper circulation. It is treated with defibrillation, which applies an electric shock in an attempt to reset the heart rhythm. As the high energy of this shock risks long-term harm to the patient, means of reducing it without compromising treatment efficacy are of great interest. One approach to maintaining efficacy is to improve the success rate of such low-energy shocks (i.e., pulses) through the proper timing of their application as defibrillation protocols, which consist of one or more pulses with predetermined inter-pulse periods. In practice, however, the effects of application timing remain to be tested for any of the multi-pulse protocols proposed in literature. We use (de)fibrillation simulations to show that such timing matters: The success rate of single-pulse protocols can vary by as much as 80 percentage points depending on timing, and using more shocks in succession only lessens this sensitivity up to a point. We also present evidence that feedback-based defibrillation on a shock-by-shock basis may be the only practical means of using timing to increase treatment efficacy, as we also generally find any optimal application timings to be specific to each combination of protocol and fibrillation episode.

## 1 Introduction

Ventricular fibrillation is a heart condition of high global mortality (Mehra, 2007; Rappel, 2022). Fibrillation occurs when the electric signal responsible for the coordination of the heart’s pumping mechanism is disrupted and thus impedes blood circulation. Such disruptions are most dangerous when they occur in the ventricles, the largest chambers and pumping-action contributors of the heart; without their contractions, circulation ceases almost entirely and sudden cardiac death follows shortly after.

The contemporary treatment of fibrillation, defibrillation, relies on a strong electric shock (Ammannaya, 2020; Cheskes et al., 2022) with potentially harmful side effects due to the high currents it induces in a patient’s body. These side effects include:

• tissue damage (Xie et al., 1997; Tereshchenko et al., 2009),
• traumatic pain (Godemann et al., 2004; Marcus et al., 2011), and
• worsening prognosis (Mackenzie, 2004; Poole et al., 2008; Moss et al., 2012).

This overall risk profile motivates the development of less intrusive defibrillation protocols, as long-term consequences can even include increased susceptibility to further fibrillation episodes in the future.

Treating fibrillation with weaker, low-energy shocks (i.e., pulses) would lessen the risk of harmful side effects. Given proper application, such pulses have been shown to be capable of terminating fibrillation in simulations of cardiac-tissue electrophysiology (Lilienkamp and Parlitz, 2020; Garzón and Grigoriev, 2024). This theoretical efficacy of pulses has been linked to the transiently chaotic nature of fibrillation: It moves and changes shape erratically, yet will often end of its own after some time (Strain and Greenside, 1998; Lilienkamp et al., 2017; Lilienkamp and Parlitz, 2018; Aron et al., 2019; Rappel et al., 2022). While this self-termination process usually takes far too long to be considered a viable means of “treating” fibrillation, its existence does suggest that it should indeed be possible to effectively force fibrillation toward timely (self-)termination through proper application of external perturbations (e.g., pulses) (Lilienkamp and Parlitz, 2020).

Viable low-energy defibrillation protocols that match the overall efficacy of traditional defibrillation have yet to be found, but many of the protocols proposed so far feature multiple pulses in place of just one (Fenton et al., 2009; Luther et al., 2011; Janardhan et al., 2014; Buran et al., 2017; Otani et al., 2019; Buran et al., 2022; Lilienkamp et al., 2022b; Aron et al., 2023; Buchan et al., 2023). Using multiple pulses offers more freedom in protocol design, and thus possible avenues for raising treatment efficacy at even lower pulse strengths. The time periods between these pulses can either be of some set length(s) or tailored to the fibrillation episode through some feedback mechanism. Research into such mechanisms to control fibrillation is ongoing, as feedback-based defibrillation would obviate the difficult task of having to find some universally applicable period length(s) (Buran et al., 2023; Suth et al., 2024); these efforts also extend to experiments on actual cardiac tissue, as is the case with (e.g.,) optical approaches (Hussaini et al., 2021; Diaz-Maue et al., 2024).

It remains unclear to what degree proper application timing could help improve the efficacy of multi-pulse defibrillation protocols with set inter-pulse periods. Although such multi-pulse approaches are commonly proposed in research, the effect of proper timing has yet to be quantified for anything beyond basic single-pulse defibrillation (Steyer et al., 2023). This state of affairs leaves potentially substantial defibrillation performance gains on the table—gains which could help narrow the efficacy gap between low-energy and traditional fibrillation protocols further.

In this paper, we use simulations of fibrillating cardiac tissue to test and quantify the timing sensitivities of various multi-pulse defibrillation protocols with set inter-pulse periods. To that end, we first go over our choice of cardiac-tissue model and how we implement it for straightforward, rapid evaluation on multiple processors in parallel. We then discuss our defibrillation model, which extends the functionality of our basic fibrillation simulation in a manner which does not compromise program parallelisability. In the context of this defibrillation model, we also introduce our protocols of interest, along with our method of measurement for their timing sensitivities. Lastly, we show, discuss, and then interpret our timing-sensitivity findings for each of these protocols in detail.

## 2 Cardiac-tissue model and simulation

Here, we discuss our choice of cardiac-tissue model—the Fenton-Karma model—for our (de)fibrillation simulations, and how we implement it for straightforward and rapid evaluation on multiple processors in parallel. We must rely on such computer simulations because they allow us to replicate the same fibrillation episode(s) to ensure comparability across our timing experiments, and because the large number of defibrillation attempts needed to gauge timing sensitivity would be too costly and laborious to conduct on actual tissue.

### 2.1 The Fenton-Karma model is a versatile cardiac-tissue model

Cardiac (muscle) tissue uses electric signals to coordinate its contraction and maintain a functional heartbeat. To ensure proper contraction, such signals must traverse the heart along a single direction and be robust to disruption, which is achieved by properties of its cells that are typical of excitable media: They constitute a network of excitable units and only propagate incoming signals of sufficient strength to their neighbours, and only if they have not already done so too recently; they also prevent premature signal dissipation by propagating signals at a set strength, ensuring full coverage of the heart from an initial stimulus. All these robustness-enhancing properties of the heart’s muscle cells, however, can also promote self-sustained signal disruption (e.g., fibrillation) under the right conditions, resulting in cardiac arrest due to insufficient contraction strength and circulation.

Cardiac tissue models can be demanding to simulate depending on how closely they model the physiology of the individual muscle cells and their signal-propagation mechanism (Bittihn, 2015; Alonso et al., 2016; Rappel, 2022). This mechanism involves the coordinated exchange of various ion species (i.e., currents) between each cell and its environment, changing (and then restoring) its transmembrane voltage in the process. This exchange is mediated by time-dependent permeabilities of the cell’s membrane to each ion species; these, along with the currents they control, can be modelled individually or in aggregate(s). Depending on how this is done, a given cardiac-tissue model can feature anywhere between 2 and 20+ differential equations, along with an arbitrary number of auxiliary equations (Fenton and Cherry, 2008).

**FIGURE 1:**
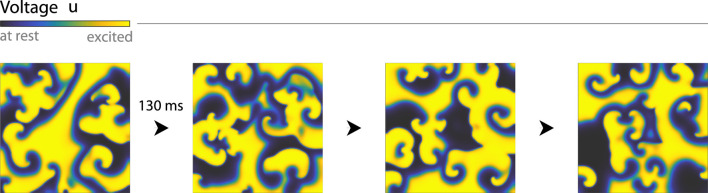
The contractions of the heart coincide with the excitations of its muscle cells, which are visible in their transmembrane voltages (image colours). Excited cells propagate contraction signals to any neighbours sufficiently close to rest, sustaining the fibrillation present in this simulated Fenton-Karma model of a thin sheet of cardiac tissue. The resulting fibrillation patterns are chaotic and change shape rapidly, which is also evident in the model’s dominant time-scale of only ∼ 130 ms (or ∼ 7.7 Hz).

The (dimensionless) Fenton-Karma cardiac-tissue model is designed to be mathematically simple and straightforward to implement in simulations (see Figure 1), yet versatile in behaviour (Fenton and Karma, 1998). The model features a reaction-diffusion equation for the transmembrane voltage u , as well as ordinary differential equations for two (aggregate) ion permeabilities v , w :

$$\partial_{t}u=\nabla \cdot \left(D\;\nabla u\right)-I_{total}\left(u,v,w\right)/C_{m},$$ (1)

$$\begin{array}{ll} \partial_{t}v & =H\left(u_{c}-u\right)\;\left(1-v\right)/{\tau_{v}}^{-}-H\left(u-u_{c}\right)\;v/{\tau_{v}}^{+},\\ \text{and}\;\partial_{t}w & =H\left(u_{c}-u\right)\;\left(1-w\right)/{\tau_{w}}^{-}-H\left(u-u_{c}\right)\;w/{\tau_{w}}^{+}. \end{array}$$

Here, $I_{total}$ is the net (ion) current flowing into a given muscle cell and H the Heaviside step function. This minimal model can emulate more complex ones through proper adjustment of its 14 parameters, providing great versatility at low computational cost. We use the same parameter values as Lilienkamp et al. (2022b) at a numerical tissue size of $150^{2}$ pixels with no-flux boundary conditions.

### 2.2 Parallelisation allows for (de)fibrillation simulation on large scales

We ensure both high evaluation speed and ease of implementation for our (de)fibrillation simulations by restricting them to simple numerical-integration and boundary-condition algorithms:

• “Forward in time, centred in space” (FTCS) for the reaction-diffusion equation (Roache, 1976),
• the “ghost-point method” for the (no-flux) boundary conditions (Smith, 1985),
• a nine-point stencil for the diffusion operator (Lynch, 1992), and
• “Rush-Larsen” for any permeabilities (MacLachlan et al., 2007).

These algorithms trade numerical accuracy for speed, being either first- or second-order methods at most. Such trade-offs are common in (de)fibrillation simulations, as any gains in numerical accuracy come with a disproportionate loss in speed (Bittihn, 2015). We do, however, use the marginally slower nine-point diffusion stencil instead of the standard five-point one to prevent directional biases due to unevenly distributed numerical errors in our simulated tissue, which could skew (de)fibrillation behaviour otherwise.

We run our simulations on multiple graphics processing units (GPUs) in parallel for further gains in evaluation speed at little additional program complexity. This is possible due to the simplicity of our chosen numerical algorithms, which only consist of elementary matrix operations that are straightforward to divide between multiple processors. This simplicity, in turn, allows for easy implementation on GPUs that each contain thousands of processors with vast amounts of shared memory at a very high level of overall performance. We implemented these GPU programs with the CUDA Fortran programming language, which supports parallelised matrix operations (and many others) out of the box (Ruetsch and Fatica, 2013).

## 3 Defibrillation model and timing

Here, we discuss how we can simulate the application of defibrillation protocols to fibrillation tissue in a way that is compatible with program parallelisation for fast evaluation. We also cover the five protocol types of interest to us (which differ in their inter-pulse periods) and how we gauge their timing sensitivities by simulating five representative protocols of each of these types with 1001 different timings.

### 3.1 Defibrillation protocols can be modelled through the currents they induce

Defibrillation is a complex physiological process that begins with the application of an external electric field of set strength to fibrillating cardiac tissue. This field induces a strength-dependent number of new currents (i.e., contraction signals) at sufficiently large conductivity heterogeneities (e.g., scars or vessels) present throughout the tissue. In this way, these heterogeneities act as virtual electrodes which disturb and may overwhelm the fibrillation present (Cheng et al., 1999; Pumir et al., 2007; Luther et al., 2011; Bittihn et al., 2012; Bittihn, 2015; Efimov et al., 2000). If the fibrillation is stopped in time, the defibrillation is a success and proper contractions can resume.

Simulating the full defibrillation process would increase program complexity and make rapid, large-scale evaluation effectively impossible. To achieve such fidelity, we would need to add some conductivity heterogeneities and their corresponding (field-dependent) boundary conditions to the tissue. These boundary conditions would each require individual processing at every simulation step, much to the detriment of program parallelisability. The added heterogeneities would also impose strict resolution requirements on the simulation, as they must be well-resolved to properly induce new currents in response to simulated electric fields. Using such a resolution, in our experience, increases the required computational workload ten- or even hundred-fold beyond that of a basic fibrillation simulation.

We avoid the complexity of the full defibrillation process by modelling only the net effect(s) of defibrillation protocols on cardiac tissue instead of the entire chain of events: Each protocol is represented by the average current it induced across all conducitivity heterogeneities through its electric field over time. We can apply this average current in our simulations by adding it to the cardiac-tissue model’s net current $I_{total}$ (Equation 1) at specific induction sites of random location and conductivity, which we place until they cover a set fraction ( ∼ 25%) of the tissue at a given size ( $2^{2}$ pixels). This process emulates the effects of the essentially random locations and sizes of conductivity heterogeneities in actual defibrillation and removes the need to simulate the electric field, the heterogeneities, and the interactions between the two.

Modelling defibrillation protocols through their average induced current(s) can emulate them in their number and frequency of pulses only, as we cannot actually measure the strengths of the individual currents induced by their electric fields. Instead, we first associate every field activation (i.e., pulse) of a given actual protocol with a rectangular current profile of some height (i.e., pulse strength) and width (i.e., pulse length), which depend on the activation amplitude and duration of the field, respectively. We then postulate values for these heights and widths without measuring actual field-induced currents; this effectively creates a current profile of a protocol with a known number and periodicity of field activations, but unknown amplitude(s) and duration(s).

**FIGURE 2:**
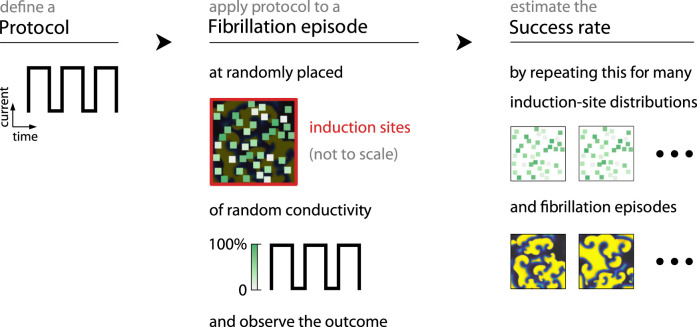
We estimate the success rate of a given defibrillation protocol is in three stages: We define a current profile to represent the average current induced by the electric field of its electric field (left), apply this profile to a fibrillation episode at randomly generated induction sites of varying conductivity (centre), observe the outcome, and then repeat this process over multiple induction-sites distributions as well as fibrillation episodes (right) to count the ratio of successful defibrillations among them.

We estimate the defibrillation success rate of a defibrillation protocol by applying its current profile to multiple fibrillation episodes at various induction-site distributions (see Figure 2), as both influence the defibrillation outcome. For each such sample, we apply the numerical protocol in question to a given episode at induction sites placed according to a given distribution and observe the outcome over ten characteristic time scales of the underlying cardiac-tissue model ( ∼ 1.3s): If the peak transmembrane voltage across the tissue drops under the excitation threshold of the model ( ∼ 0.1a.u.), further self-sustained fibrillation is impossible and the attempt considered a success. Counting the successes across all sampled episodes and induction-site distributions then gives us the protocol’s defibrillation success rate. We sample every protocol over $10^{4}$ such combinations of episodes and induction-site distributions to ensure a uniform (maximum) standard error of one percentage point in all our success-rate estimates: Given a set number of fibrillation episodes, we adjust the amount of site distributions as needed to reach this total sample size.

### 3.2 There are three general protocol types to emulate and test

**FIGURE 3:**
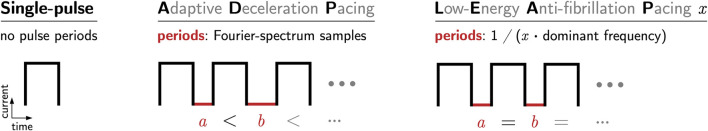
Each defibrillation protocol is represented by the average current it induces in fibrillating cardiac tissue over time. Our three general protocol types of choice each feature pulses of uniform strength and length, but differ in the lengths of their inter-pulse periods (red): Widening for ADP, uniform for LEAP.

There are five protocol types of interest to us, which are each based on one of three general types (see Figure 3). These three general types all feature uniform pulses (i.e., field activations) of arbitrary strength and length with specific, set inter-pulse periods:

- 1x **Single-pulse defibrillation** (see Figure 3, left) usually involves a very potent pulse (i.e., shock) and is closest to what is used in contemporary medicine.
- 1x **Adaptive Defibrillation Pacing** (ADP; see Figure 3, centre; Lilienkamp et al., 2022b) uses multiple pulses with increasing period lengths. These inter-pulse periods are determined by sampling the various time scales observed in fibrillation through its Fourier spectrum.
- 3x **Low-Energy Anti-Fibrillation Pacing** x (LEAP; see Figure 3, right; Luther et al., 2011)uses multiple pulses at a uniform period. This period is typically the inverse of the dominant frequency of fibrillation ( ∼ 7.7 Hz for our cardiac-tissue model) scaled by some speed factor x . We use three different values for this speed factor (0.5, 1.0, and 1.5) for three LEAP types in total.

We split each multi-pulse protocol type (ADP and LEAP) into separate five- and ten-pulse variants. This allows us to gauge the influence of pulse count on defibrillation performance and timing sensitivity, especially in direct comparison with single-pulse defibrillation.

### 3.3 Timing sensitivity is measured through representative protocols

**FIGURE 4:**
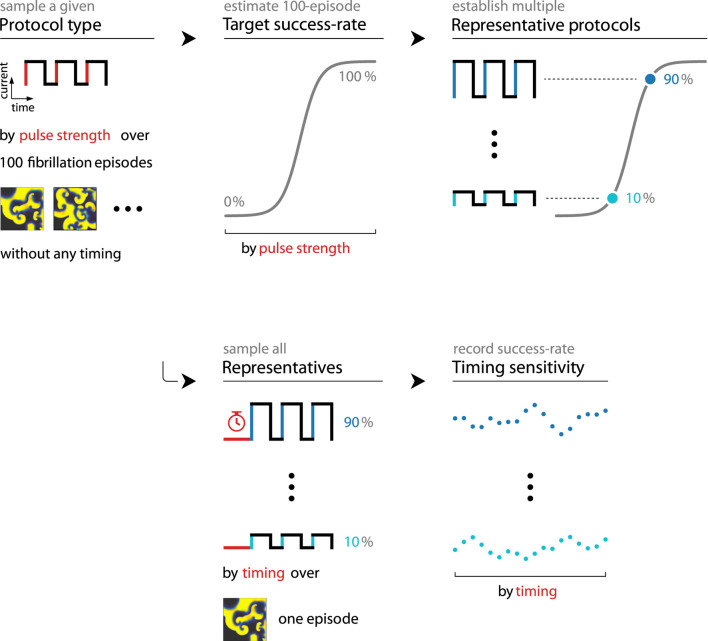
We measure the timing sensitivity of a given protocol type and pulse count in two stages (rows): First (top), we establish five representatives of known success rates when applied to 100 fibrillation episodes without any timing, ensured through appropriate uniform pulse strengths. Then (bottom), we apply these representatives to copies of the same episode at various timings and record their new success rates.

**FIGURE 5:**
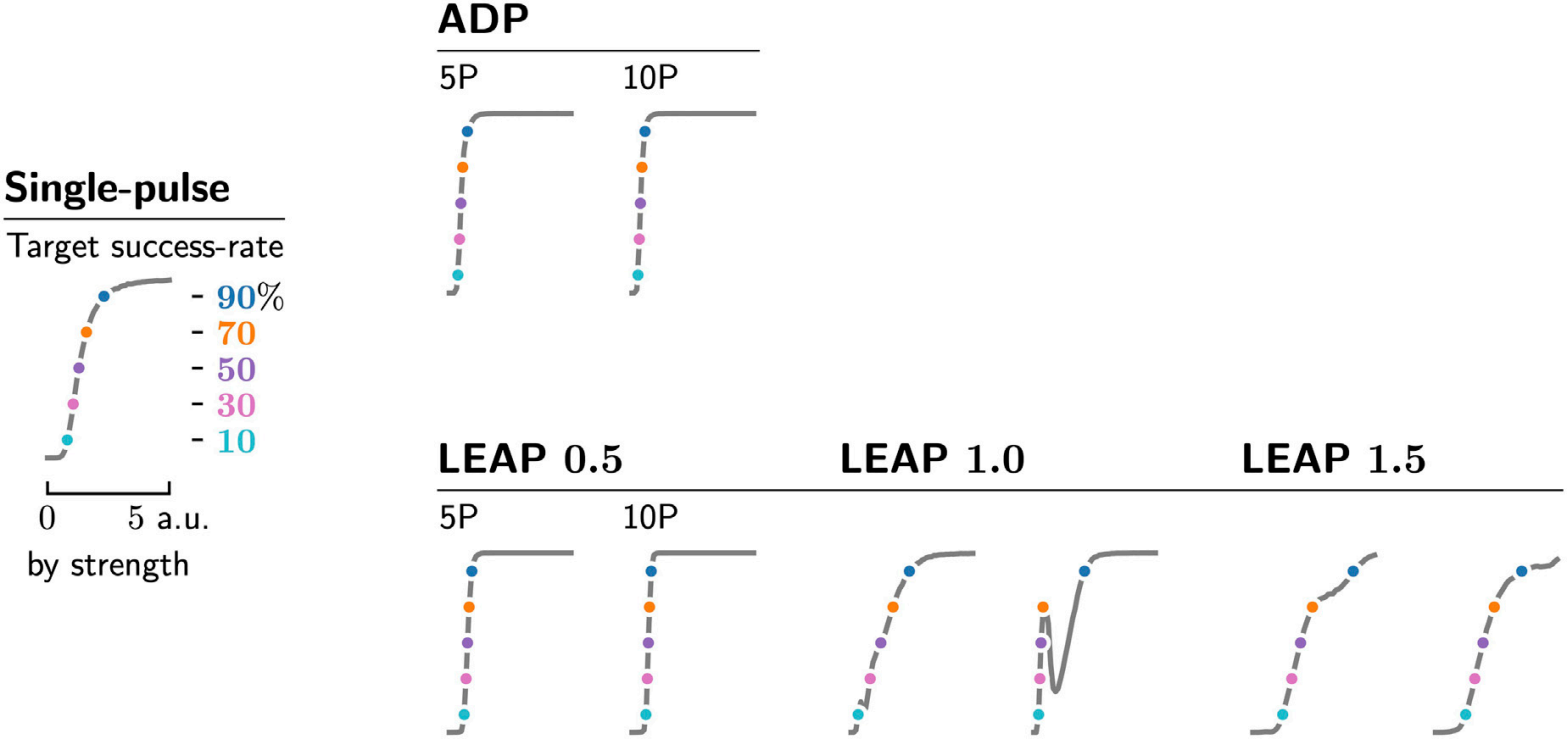
Each protocol type (rows and cols., bold names) and pulse count (cols., pairs) is represented by five protocols of known success rates (y-axes, coloured dots) when applied to 100 fibrillation episodes without any timing considerations. We ensure this by selecting appropriate values for their uniform pulse strengths (x-axes, coloured dots), which we determine by sampling each protocol type and pulse count over pulse strengths ranging from 0 to 5 a.u. (x-axes) and recording the corresponding averaged success rates (y-axes) shown as dose-response curves (grey). The non-monotonic LEAP 1.0 and 1.5 dose-response curves shown here are in line with previous findings on dose-response curves of multi-pulse protocols, where it was found that their curves may assume more complex shapes (Lilienkamp et al., 2022a).

We establish five representatives of each protocol type and pulse count in preparation for the measurement of their timing sensitivities (see Figure 4, top), as the pulse strengths and lengths of their current profiles are free parameters that we need to define without a clear best choice for either. To avoid fully arbitrary choices, each representative protocol has a set defibrillation success rate equal to one of five target values (namely, 10, 30, 50, 70, or 90%) when applied to 100 fibrillation episodes without any timing; we enforce each such target by choosing an appropriate pulse strength at a set pulse length of 2 ms (see dose-response curves in Figure 5). These representatives of thus known (average) reliability also provide a convenient basis of comparison for any timing-related effects we find.

Timing sensitivities of our protocol types and pulse counts were measured by applying their representative protocols to copies of a specific fibrillation episode at various initial timings (see Figure 4, bottom). These timings range from 0 to 10 s at 1001 samples in total, giving us 1001 defibrillation success rates per representative. Measuring these (likely episode-specific) success rates with copies of the same fibrillation episode ensures comparability across all representatives, protocol types, and pulse counts. These measurements should thereby allow us to compare the timing sensitivities of all protocol types and pulse counts through their respective representative protocols.

## 4 Results and discussion

Here, we discuss the three main observations we can make based on our timing-sensitivity measurements: All protocols tested are timing-sensitive, sensitivity decreases as the number of pulses rises, and any optimal timings we see are generally specific to each protocol type and pulse count.

### 4.1 All protocols show timing sensitivity

**FIGURE 6:**
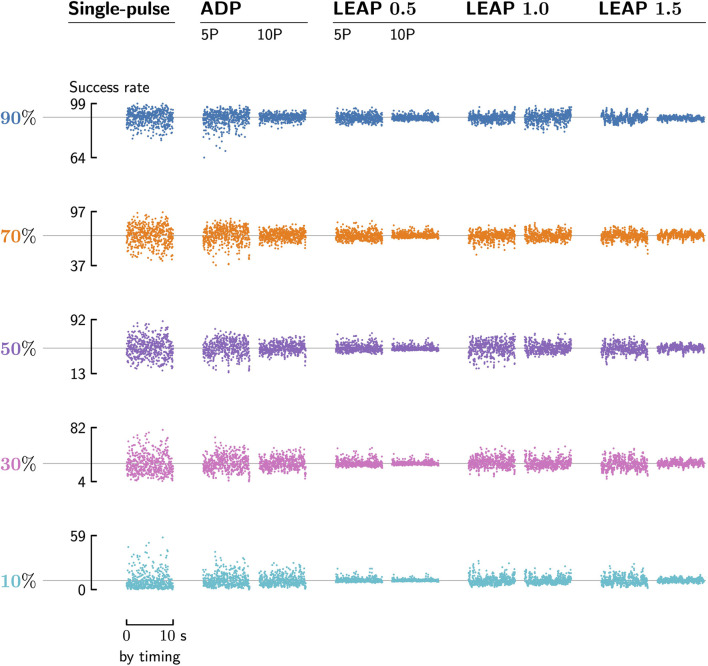
All protocols show timing sensitivity in their defibrillation success rates (y-axes), measured by applying each of them to copies of the same fibrillation episode at various timings (x-axes). This holds across protocol types (cols., bold names), pulse counts (cols., pairs), and pulse strengths (rows). We choose the pulse strengths such that a given protocol reaches a specific target success-rate (rows, colours and horizontal lines) when applied to 100 different episodes without any timing considerations.

We find timing sensitivity in the defibrillation success rates of all protocol types and pulse counts (see Figure 6). Sensitivity is highest in single-pulse protocols (see Figure 6, col. 1), where success rates can vary by as much as 80 percentage points depending on timing; even the least sensitive protocols still see fluctuations well within 10–20 percentage points. If somehow leveraged, these fluctuations could deliver substantial gains (or losses) in defibrillation reliability given (im)proper application timing.

The average of all defibrillation success rates taken over all timings always matches the target success-rate of a given protocol, which is measured over 100 different fibrillation episodes without any timing influence (see Figure 6, horizontal lines). This is due to fibrillation being chaotic in both space and time: Any correlations in it decay exponentially in both space and time as well, and waiting a short period produces a new, unrelated episode as far as success-rate averaging is concerned; it thus makes no difference whether we average the success rate (or similar observables) over long periods of time or different episodes.

Protocols with a target value of 50% for their defibrillation success rate without any timing (see Figure 6, row 3) are most affected by timing, while those closer to 0% and 100% are less sensitive to its effects. This is expected, as defibrillation attempts are pass-or-fail trials; accordingly, they show the highest possible variance at a success rate of exactly 50%, and the smallest variance at either 0 or 100%.

### 4.2 The more pulses, the less timing-sensitive the protocol

Protocols with more pulses are usually less timing-sensitive than those with fewer pulses of the same type (see Figure 6, cols., pairs). This is also the case when we compare any multi-pulse protocols with the single-pulse ones (see Figure 6, col. 1). The decrease in timing sensitivity with more pulses is in line with the idea that a given protocol can be viewed as a sequence of independent pass-or-fail trials (i.e., pulses), where a larger number of trials would increase their combined success rate while reducing the variance at the same time. This view is a simplification, however, as consecutive pulses have at least some correlated effect(s) on the underlying fibrillation when applied in quick succession.

While generally true within our data, the above observation has one notable exception: Despite having twice the pulse count, the ten-pulse LEAP 1.0 protocols (see Figure 6, col. 5) show a negligible difference in timing sensitivity compared to their five-pulse counterparts (see Figure 6, col. 4). We currently do not know why only this particular protocol type violates what appears to be an otherwise general trend.

When comparing protocols of different types, a higher pulse count need not imply less timing sensitivity. For example, the ten-pulse LEAP 1.0 protocols (see Figure 6, col. 7) are overall more timing-sensitive than both the five-pulse LEAP 0.5 and 1.5 protocols (see Figure 6, cols. 4 and 8), respectively.

### 4.3 Protocols generally do not share the same optimal timings

**FIGURE 7:**
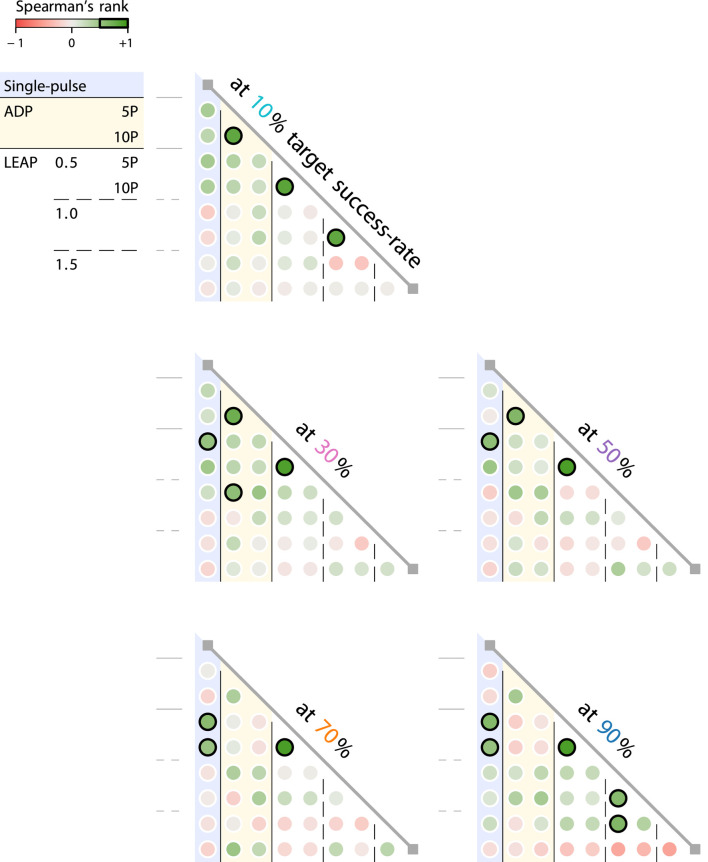
Of all protocol types/pulse counts (matrix rows and cols.), only the five- and ten-pulse LEAP 0.5 protocols (matrices, row 5 and col. 4) allow for the mutual inference (bold, green circles) of optimal defibrillation timings across all target success-rates (matrices). For every other potential pair, there is at least one target success-rate (matrix) showing an insufficient timing-correlation ( < 0.5, thin circles) between them. We measure these correlations through Spearman’s rank, as it makes no assumption beyond monotonicity (e.g., linearity) about the relationship between the timing data.

We generally cannot infer optimal defibrillation timings from other protocols of a different type and/or pulse count. Doing so would require consistent timing-sensitivity correlations between all their respective representative protocols of equal target success-rate, which is only the case between the five- and ten-pulse LEAP 0.5 protocols (see Figure 7). We quantify these correlations through Spearman’s rank, which tests for a monotonic relationship between success-rate time series of any two protocols by computing the Pearson correlation of their ranks (Myers et al., 2010). The lack of consistent correlations of this kind implies that there does not exist any universally optimal timings intrinsic to a given fibrillation episode that we might hope to detect and leverage for all protocols.

We can infer optimal defibrillation timings from other protocols of different pulse strengths if they are of the same type and pulse count. If they are, the peaks and valleys of their timing sensitivities tend to align well (see Figure 6, cols.). This alignment is most evident among single-pulse and ADP protocols (see Figure 6, cols. 1-3), respectively. This observation, however, is not particularly novel: We would expect borderline identical protocols to interact with a given fibrillation episode in similar ways, resulting in similar optimal timings.

## 5 Conclusion

The application timing of defibrillation protocols with set inter-pulse periods affects treatment efficacy, but may be unfeasible to leverage in practice because optimal timings are unique to each protocol and fibrillation episode. The erratic behaviour and short time-scale of fibrillation would already put immense time constraints on such timing decisions, assuming we had a readily measurable observable capable of predicting the optimal timings of all multi-pulse protocols. No such observable exists, however, as it would have to somehow correlate with all our generally uncorrelated timing-sensitivity measurements.

Defibrillation protocols with variable, feedback-driven inter-pulse periods may be the only feasible means of leveraging timing for improvements in treatment efficacy. This is because every pulse changes the already erratic fibrillation present, to the point where consecutive pulses each interact with very different fibrillation states. It thus stands to reason that each pulse ought to be timed individually to maximise the efficacy of a given protocol, essentially treating it as a sequence of single-pulse protocols. Suitable observables for predicting these optimal single-pulse timings may exist, as we can only rule out the existence of such an observable for general multi-pulse protocols based on our findings.

The results of this study are limited in both scope and general applicability—especially in the broader medical context—due to some simplifying assumptions we have made in its design: We

• simulate only homogeneous and isotropic cardiac tissue,
• consider only two-dimensional sheets of infinitely thin cardiac tissue,
• model defibrillation through local current injection without an explicit electric field,
• use a single generic (i.e., non-ionic) cardiac-tissue model instead of a more sophisticated one, and
• disregard all interactions between the electric signal and the resulting mechanical contractions.

Chiefly, these assumptions make it difficult to translate any of the protocols we use in simulations into ones that can be applied in actual experiments. The root of this difficulty is the defibrillation model, which disregards the electric field entirely and thereby loses the link between the induced currents and their cause; this, in turn, leaves us with no straightforward way of inferring the necessary field strengths and durations to replicate any protocols used in this study for use on real cardiac tissue.

With the limitations of our findings in mind, future work could attempt to reproduce our results with simulations based on different cardiac-tissue and/or more realistic defibrillation models. Further research may also help identify possible observables for the prediction of optimal single-pulse timings in feedback-based defibrillation protocols; in fact, some viable candidates have already been proposed (Buran et al., 2023; Suth et al., 2024). Conceivably, using these predictive observables will require some sophisticated data analysis (Datseris and Zelko, 2024); they could also incorporate input(s) from other parts of the cardiovascular system in a practical application of network physiology.

## Data Availability

The datasets presented in this article are not readily available because because they consist of a very large amount of simulated data. Requests to access the datasets should be directed to ulrich.parlitz@ds.mpg.de.
